# The Effects of Khat Chewing among Djiboutians: Dental Chemical Studies, Gingival Histopathological Analyses and Bioinformatics Approaches

**DOI:** 10.3390/bioengineering11070716

**Published:** 2024-07-15

**Authors:** Fatouma Mohamed Abdoul-Latif, Ayoub Ainane, Ali Merito, Ibrahim Houmed Aboubaker, Houda Mohamed, Sanaa Cherroud, Tarik Ainane

**Affiliations:** 1 Center for Research and Study of Djibouti, Medicinal Research Institute, Djibouti BP 486, Djibouti; 2 Superior School of Technology, University of Sultan Moulay Slimane, BP 170, Khenifra 54000, Morocco; 3 Peltier Hospital of Djibouti, Djibouti P.O. Box 2123, Djibouti

**Keywords:** dental pathologies, gum and tooth pathology, morphological analysis, *Catha edulis*, chewing

## Abstract

This study examined the effects of khat chewing on oral gingival conditions by adopting a targeted process which combined physicochemical analyses of the teeth, histopathological examinations of the gums, and bioinformatics modeling. The physicochemical evaluation of teeth in khat consumers compared to non-consumers was carried out using specific analytical techniques; hence, the results of this initial investigation revealed significant erosion of the tooth enamel due to khat chewing, as well as an alteration of the essential chemical composition of the teeth. Additionally, the histopathological analyses complemented preliminary studies by showing severe inflammation of the gums and oral mucosa in khat users. The understanding of these studies was enriched by bioinformatics analysis, where modeling was carried out via computational methods. This analytical phase examined molecular docking mechanisms, including the interaction between cathinone, the main alkaloid of khat, and the protein receptors involved in the protection of gingival tissues against infections. In summary, this multidisciplinary research provided an in-depth view of the oral health issues related to khat chewing, combining experimental studies with bioinformatics perspectives.

## 1. Introduction

*Catha edulis* Forsk, commonly known as Khat or Qat, is a plant native to the Arabian Peninsula and parts of East Africa, belonging to the Celastraceae family [1]. The traditional practice of khat chewing is rooted in the ancient cultures of these regions, where it is seen as a way of maintaining food traditions while adapting to local environments. This evolution of consumption patterns represents an important continuity in the cultural tradition of these regions, particularly in Djibouti, where a large part of the population uses khat to improve their mental and physical well-being. Khat-derived products are produced with the aim of stimulating the physical and mental abilities of consumers [2,3,4].

The leaves of this shrub contain psychoactive substances that produce effects such as sympathomimetic and euphoric sensations [5]. The primary active compound, cathinone, bears structural similarities to amphetamines, making it the most prominent ingredient [6]. Further analysis of other leaf constituents has revealed the presence of phenylalkylamine compounds, including cathine and beta-cathinone, which also share notable similarities with amphetamines. Additionally, these leaves are rich in tannins, vitamins, minerals, and flavonoids, which are present in significant proportions [7,8].

Similar to any botanical organism, khat exhibits a range of positive and negative effects [9]. Advocates of khat chewing point out various health advantages, such as its potential to lower blood sugar levels, its use as an asthma remedy, and its ability to alleviate symptoms associated with intestinal tract disorders [10]. However, there are emerging drawbacks indicating that khat consumption carries health risks, has detrimental effects on various aspects of life, and results in medical consequences as well as socio-economic repercussions [11].

In the field of medical scientific research, the exploration of the properties of this plant remains limited, although some researchers highlight its potential therapeutic properties and consider its applications in the treatment of various conditions [12]. Others, however, issue warnings regarding the associated risks, highlighting possible adverse effects on the cardiovascular system, the central nervous system, as well as potential implications for mental health [13]. As for the toxicity of khat, no definitive link has yet been established between a specific mechanism and the toxic effects of this plant. Existing studies have primarily focused on the toxicity associated with cathine and other phytochemicals [14]. However, previous research has highlighted a notable characteristic of khat: its ability to produce antioxidant agents [15]. Although traditionally considered beneficial, antioxidants may pose potential risks under certain circumstances or at high concentrations [16].

This study aims to examine the oral conditions influenced by khat chewing. Three main areas of research are thus addressed: (1) the physicochemical characterization of the teeth of individuals consuming khat; (2) an analysis of the effects of khat on gingival plasma cell infections in consumers; and (3) a bioinformatics approach targeting cathinone as the predominant compound, based on energetic studies and involving the key proteins present in gingival tissues, thereby contributing to protection against oral gingival infections.

## 2. Material and Methods

### 2.1. Physicochemical Analysis of the Teeth

#### 2.1.1. Sampling of Human Teeth

**Figure 1:**
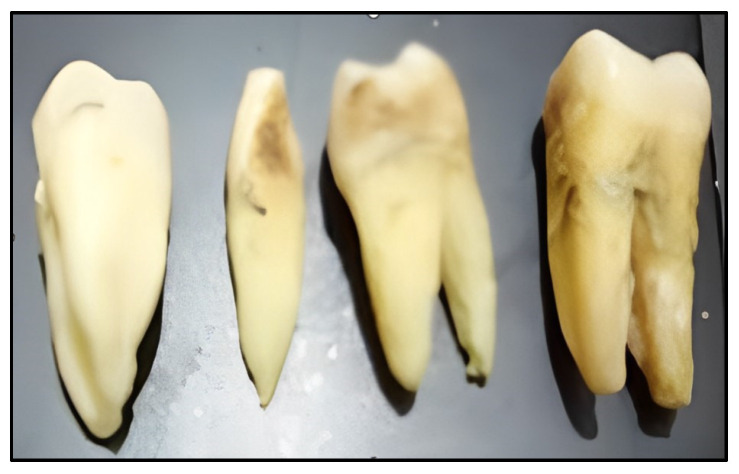
Tooth samples from Khat consumers.

As part of the first step of the study, which concerns the physicochemical analysis of the teeth of Khat consumers, samples were taken from adult human teeth collected from traditional practitioners working in the field of traditional medical approaches in Djibouti—in the Tadjourah region, precisely (Figure 1). These samples were subjected to verification by a specialist doctor from the Peltier hospital in Djibouti. Subsequently, the extracted teeth were isolated and divided into two groups based on the frequency of khat consumption by the individuals. The teeth of subjects who consumed khat once or twice a week for five years were grouped into the category of khat users (DK), while those who never consumed khat were classified as non-users (DNK). The condition of each tooth in each group was assessed. Before any analysis, the teeth were thoroughly cleaned under a stream of running water using a soft-bristled toothbrush. A surgical scalpel was used as necessary to remove limescale. Subsequently, the teeth were rinsed and preserved in distilled water at room temperature. During the analysis, the human tooth samples were analyzed either in block forms or they were ground into powder (whole tooth) according to the specified analysis methodology.

#### 2.1.2. Analysis Techniques

To characterize the teeth physicochemically, several analysis procedures were implemented. X-ray diffraction (XRD) was performed with Bruker XRD model D8-Advanced equipment. This technique makes it possible to determine the crystal structures of the samples. The recorded diffraction patterns benefited from the geometric precision offered by the Bragg–Brentano model, which allowed for the detailed analysis of the crystal structures present in the teeth. In parallel, Fourier transform infrared (FTIR) spectra were acquired to examine the molecular properties of the samples. This analysis was carried out in the range of 400–4000 cm^−1^ by amalgamating 1 mg of sample powder with 30 mg of KBr. The infrared spectrometer used was a NICOLET NEXUS 470, equipped with a temperature-adjustable SPECTRA TECH DRIFT cell, allowing for the precise analysis of chemical bonds and molecular vibrations. For the morphological study of the samples, scanning electron microscopy (SEM), coupled with X-ray energy dispersive spectroscopy (EDS), was used. The microscope employed was a Jeol 5600LV, operating at a voltage of 30 kV and a pressure of 20 Pa. This configuration made it possible to reveal the chemical composition of the samples without requiring the use of a thin layer for SEM, thus facilitating the direct observation of tooth surfaces. A microhardness analysis was performed on 10 samples, including khat chewers and non-chewers. The Vickers hardness index (VHN) was measured using a digital microhardness tester, allowing the hardness of teeth from different groups to be compared. Finally, thermogravimetric (TGA) and differential calorimetric (DSC) analyses were carried out to examine the thermal properties of the samples. For this, 2.0 g of powder were introduced into the DUPONT model 910 equipment. Temperature-programmed desorption (TPD) tests were carried out using an argon flow, thus making it possible to understand the thermal behaviors and the transformations of materials under the effect of heat.

### 2.2. Biological Test of Affected Tissues in the Oral Gums 

**Figure 2:**
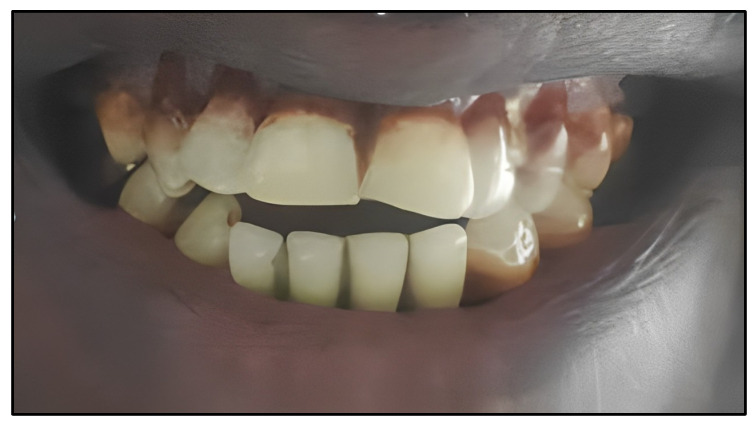
Volunteer’s teeth for histological examination.

For this second phase of our research, a public call was initiated in Djibouti to recruit volunteers meeting the inclusion criteria. Dozens of individuals responded positively. The study participants were chronic users of khat, having practiced this habit for over five years and exhibiting oral gingival alterations. All participants were fully informed about the study objectives and provided consent by signing appropriate documents. Following a thorough clinical examination and initial screening, a 38-year-old man displaying symptoms of acute periodontitis was selected as the experimental subject. The entire experiment was conducted specifically on this individual (Figure 2). Histological analysis using the Hematoxylin–Eosin method (He) was performed during this phase to characterize the connective tissues in oral conditions. This process involved taking a biopsy sample, fixing it in formalin, embedding it in paraffin, cutting it into thin sections, staining with hematoxylin and eosin, followed by microscopic observation to identify and document tissue characteristics.

### 2.3. Bioinformatic Approach

Computational research was integrated as a complementary step to this study to provide in-depth theoretical insights into cathinone, the predominant molecule in khat, as well as its interaction with the key proteins present in gingival tissues that contribute to protection against infections. In this regard, two distinct approaches were used: the modeling of the molecular interactions of cathinone with the determination of remarkable energetic descriptors [17], and the study of docking of cathinone with selected proteins [18]. 

**Figure 3:**
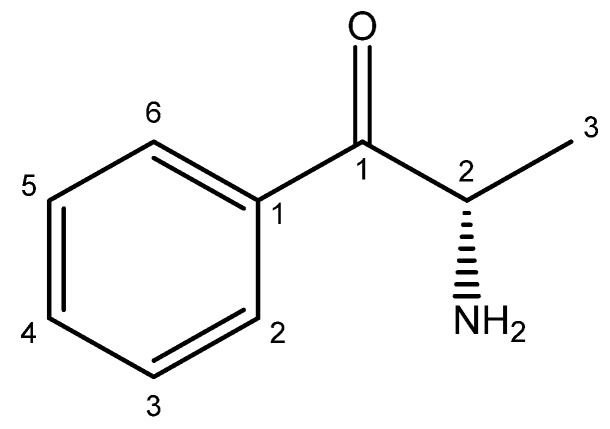
Cathinone structure (Compound CID: 62258).

For the first computational approach to cathinone (Figure 3), the MMFF94 (Merck Molecular Force Field 94) method was employed [19]. It is a set of force parameters used in computational chemistry to model molecular interactions within the framework of molecular mechanics. Various energy descriptors have been targeted for calculations, including the energy levels of upper occupied molecular orbitals (E_HOMO_) and lower unoccupied molecular orbitals (E_LUMO_), leading to other parameters such as the energy gap (E_GAP_), chemical hardness (η), electronegativity (χ), electrophilicity index (ω), and molecular flexibility (S). The formulas for calculating these global descriptors are listed in Table 1 [20]. 

In the second computational approach, docking was carried out in order to detail the mechanisms involved in the interaction between cathinone and three essential proteins present in gingival tissues. The three-dimensional structures of the proteins were extracted from the RCSB database (PDB codes: 1FD4 for defensin, 1B7S for lysozyme, and 1EGT for thrombomodulin). After optimizing the structures using the Swiss-Pdb Viewer v4.1 software, the preparation for docking required the removal of water molecules and heteroatoms, as well as the addition of polar hydrogen and the assignment of loads according to the Kollman method. Cathinone was saved in (.pdb) format and its proposed geometry was optimized through DFT calculations using the B3LYP/6-311G basis set. Docking simulations were conducted using the AutoDock Vina software 1.1.2 using a 40 Å grid in each of the x, y and z directions. The coordinates used for these simulations are listed in Table 2. Next, the BIOVIA Discovery Studio software (Studio, 2021) was employed to visualize the protein–ligand interactions, thus allowing for a detailed analysis of the interaction modes between cathinone and target proteins, and providing information on their potential as biological agents [21,22].

### 2.4. Statistical Studies

The calculations of all experimental tests were subjected to statistical evaluation using XLSTAT 2016 software (integrated with Microsoft Office EXCEL). The numerical data obtained are presented as mean ± uncertainty, with a significance level of 5%, resulting from three repetitions for each experiment, using the Student test. Furthermore, the principal component analysis (PCA) method has proven to be an essential mathematical tool for examining the correlation between various calculated parameters. The objective was to visualize and synthesize data regarding the energetic and qualitative properties of the molecular docking of cathinone with the studied proteins [23,24].

## 3. Results and Discussion

### 3.1. Teeth Characterization 

**Figure 4:**
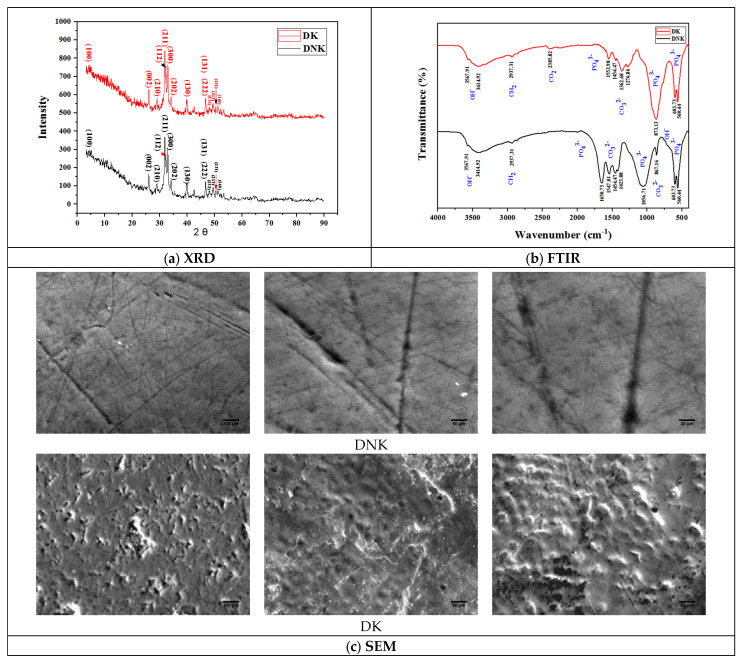

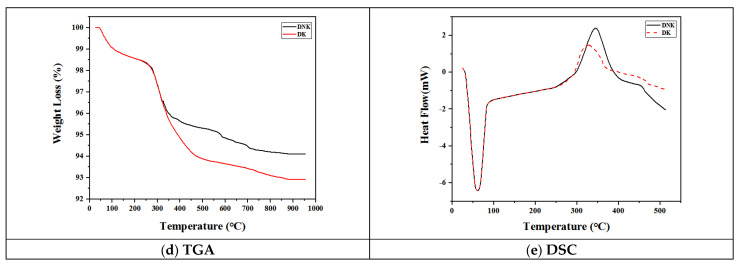
Physicochemical characterization of teeth. (**a**) XRD: X-ray diffraction. (**b**) FTIR: Fourier transform infrared. (**c**) SEM: Scanning electron microscopy. (**d**) Thermogravimetric analysis. (**e**) Differential scanning calorimetry. (DK: teeth of Khat consumers, DNK: teeth of non-consumers of Khat).

All the characteristics of the DK and DK samples, respectively, from the teeth of khat users and non-khat users, are illustrated in Figure 4a.

The utilization of the X-ray powder diffraction (XRD) method is imperative in the thorough exploration of the crystalline attributes of a substance, encompassing the constituent phases, mean crystallite dimensions, strains, crystallinity percentages, and residual stresses. The analysis of the XRD diffractograms of the (DK) and (DNK) powder specimens disclosed significant resemblances, thereby verifying the existence of hydroxyapatite (HA) in both samples, accompanied by comparable quantities of tricalcium phosphate (TCP), calcium amorphous phosphate (CAP), and octacalcium phosphate (OCP). Nonetheless, slight disparities in the (DK) sample imply the potential presence of dental pigments or organic remnants from the khat plant. The connection between hydroxyapatite and the hexagonal structure of dental enamel, known for its symmetry within the P63/m space group, has been substantiated. The lattice parameters of pure hydroxyapatite, specifically a = 9.43 Å, c = 6.88 Å, and γ = 120°, have been ascertained. A marginally more organized variant in monoclinic form has also been identified. Human tooth enamel crystals predominantly adopt a hexagonal structure, irrespective of the relative stability between the monoclinic and hexagonal phases of hydroxyapatite. Further insights were gleaned through the observation of alterations in the unit cell along the a-axis, which are linked to pH fluctuations below 5.5 units. This process, influenced by acidity, culminates in the erosion of the tooth enamel surface. Simultaneously, the amorphous CO_3_^2-^ content within the structural cavities becomes protonated, thus inducing its release from the dental environment in the form of carbon dioxide, which achieves equilibrium in the aqueous phase. Ultimately, this study furnishes numerical data that bolster the similarity of mineral compositions, while also delving into nuanced aspects potentially associated with khat consumption [25,26,27,28].

The FTIR analysis of samples (DK) and (DNK) provided additional information to the previous analysis (Figure 4b). The important results of this analysis highlight bands attributed to hydroxyapatite (HAP), particularly at 1270, 1050 and 560 cm^−1^ for PO_4_^3−^, as well as carbonate signals identified at 1550, 1380 and 875 cm^−1^. The presence of organic matter (C-H at 2935 cm^−1^) in the two samples, as well as bands linked to adsorbed molecular water (3570 and 3410 cm^−1^), are also noted. The variations in the intensity of the C-H bands attest to the differences in organic matter content between the two samples, highlighting significant distinctions [29,30].

Scanning electron microscopy (SEM) analysis of the both samples was carried out from a qualitative perspective, aiming to examine the enamel surface. This precise approach explores possible changes in dental ultrastructure induced by khat chewing. At the same time, it determines the energy dispersive surface enamel of khat chewers (DK) and non-chewers (DNK) samples (Figure 4c). SEM observations of DNK teeth reveal an almost smooth enamel layer, while those of DK khat chewers demonstrate ultrastructural alterations such as microporosities, holes, and depressions, suggestive of the early stages of bacterial caries. The quantitative analysis highlights a deterioration in microhardness and mineral content (Table 3), indicating a negative impact not only on the ultrastructure of the enamel, but also on its mechanical properties. Khat chewers showed an important decrease in calcium and phosphorus levels, suggesting a demineralizing effect. However, the Ca/P ratio increases, probably associated with the increased substitution of phosphorus by khat-derived compounds. These results highlight the detrimental influence of khat chewing on dental health, highlighting significant structural and mechanical changes [31,32,33].

The thermogravimetry (TGA) curves of the samples reveal notable differences both in terms of the amplitude of weight loss and the associated slopes. The slope of the curve for the (DK) sample is steeper than that for the (DNK) sample (Figure 4d). For the sample (DNK), a total weight loss of 6% is observed between room temperature and 900 °C, with at least four distinct slopes. The first slope indicates a rapid weight loss of about 1% from room temperature to about 100 °C, followed by a slower loss between 100 and 300 °C, then a rapid loss again between 300 and 400 °C, and finally a more progressive loss between 400 and 900 °C. For sample (DK), five distinct slopes are observed, totaling a weight loss of approximately 7%. The first slope up to 100 °C is attributed to absorbed water, while weight losses between 200 and 450 °C are related to organic matter, possibly networked water, and the decomposition of carbonate dentine. The fifth slope, observed after 450 °C, implies a possible transformation into tricalcium phosphate. Regarding the differential scanning calorimetry (DSC) method (Figure 4e), the DNK sample shows a marked exothermic peak between 300 and 400 °C, while the DK sample reveals a significant exothermic peak between 250 and 350 °C, with consistent interpretations with the weight loss profiles observed in TGA experiments [34,35,36,37,38].

In conclusion, physicochemical analyses of the teeth of khat consumers demonstrated the substantial erosion of dental enamel, a direct consequence of khat chewing. This alteration is supported by the deterioration of the crystal structure of the main dental compounds, demonstrating a decrease in tooth mass [39]. At the same time, a notable increase in organic matter was observed, likely to promote the formation of pathogenic bacterial films [40]. These results highlight the specific deleterious impacts of khat chewing on composition, crystal structure, and tooth mass, suggesting significant implications for the oral health of affected individuals.

### 3.2. Histological Test of Affected Tissues in the Oral Gums

**Figure 5:**
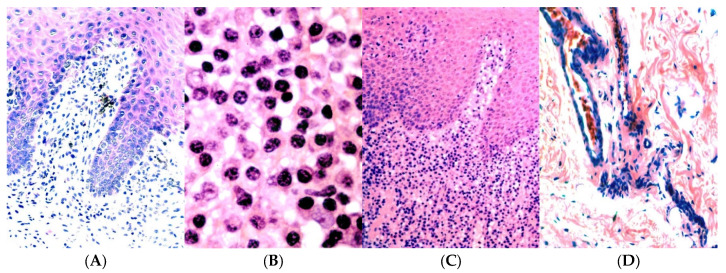
(**A**) Characteristics of connective tissue in oral lesions induced by khat. (**B**) The predominant composition of the inflammatory infiltrate consists of plasma cells (He × 400). (**C**) Gingivitis characterized by plasma cells, accompanied by hyperplastic epithelium, and a dense inflammatory infiltrate in the underlying lamina propria (He × 100). (**D**) The submucosa of chronic khat chewers exhibits fibrosis and tortuous blood vessels.

In accordance with our protocols described above, the patient was referred to a dental practitioner for a biomedical investigation of the gingival tissues due to manifestations suggestive of gingivitis in the left mandibular gingiva (Peltier Hospital of Djibouti). Despite initial periodontal treatments, such as scaling and routine oral hygiene measures, the pathology persisted for more than six months. The clinical examination revealed notable alterations on the left side of the lower gum, as well as similar abnormalities in certain areas of the upper gum. These changes were characterized by redness and swelling of the gums, particularly in the marginal and alveolar gingiva, as well as in the gingival sulcus, indicating inflammation. Similar changes were observed in the oral mucosa of the same region, with disseminated ulcers covered in fibrin, and epithelial desquamation was noticeable after drying. The patient’s general health was satisfactory, and he claimed not to take any medication. Although he could not identify the potential cause of his condition, he reported noticing swelling of regional lymph nodes over the past few months. A biopsy was performed under local anesthesia, partially removing tissue from the gingival mucosa and cheeks. A histological examination using the He method (Figure 5) revealed an erosive surface with a thickening of the epithelium, cellular exocytosis, and spongiosis. Small foci of neutrophils (microabscesses) were identified in the parakeratinized layer. No fungal filaments were detected after periodic acid-Schiff (PAS) staining. Significant expansion and intense inflammatory infiltration in the connective tissue, mainly composed of plasma cells, were observed. A histological analysis was undertaken to explore the possibility of lymphoma, but revealed a polyclonal presence of plasma cells, suggesting nonspecific inflammation. The conclusion was that the gingival and oral tissues were affected by an inflammatory pathology involving plasma cells, with potential underlying sensitization. This conclusion was based on the presence of the polymorphic infiltration of plasma cells with epithelial alterations and the absence of evidence of fungal infection. An in-depth interview with the patient revealed regular consumption of khat almost daily. It was recommended to stop this practice due to the alterations observed in the soft tissues and alveolar bone. The patient was followed at the clinic on a weekly basis, and significant improvement, with an almost complete healing of the affected gingival tissues and oral mucosa, was noted after approximately three weeks. However, despite efforts to monitor his condition, the patient refused to undergo further examinations thereafter.

Plasma cell gingivitis (PCG) is a dental condition characterized by a deep infiltration of plasma cells into the subepithelial gingival tissue [41]. Clinically, it presents as generalized erythema and edema of the gums, with a clear demarcation along the mucogingival junction [42]. Ulcerations of the affected gingival tissues are rare. The precise etiology of PCG remains undetermined, but it is often associated with an immune response to allergens, with a notable presence of plasma cells [43]. Allergens present in oral care products or food, such as khat, can trigger this reaction. Differential diagnoses include other oral inflammatory pathologies and systemic conditions, such as leukemia or lupus erythematosus [44,45]. PCG, frequently linked to khat consumption, can be explained by a complex immunological mechanism. When khat allergens come into contact with gingival tissues, they trigger an immune response. These allergens can act as antigens, stimulating the production of plasma cells, which are specialized immune cells responsible for antibody production [46,47,48,49,50]. During this process, khat allergens can penetrate the gingival tissues, where they are identified by immune cells as foreign agents. The immune cells, particularly B lymphocytes, become activated and transform into plasma cells, which synthesize specific antibodies against the khat allergens [51,52]. This immune response leads to a generalized infiltration of plasma cells into the subepithelial gingival tissue, characteristic of plasma cell gingivitis. The accumulation of plasma cells in the gingival tissues induces inflammation, clinically visible as redness and swelling of the gums [53].

### 3.3. Bioinformatic Studies

**Figure 6:**
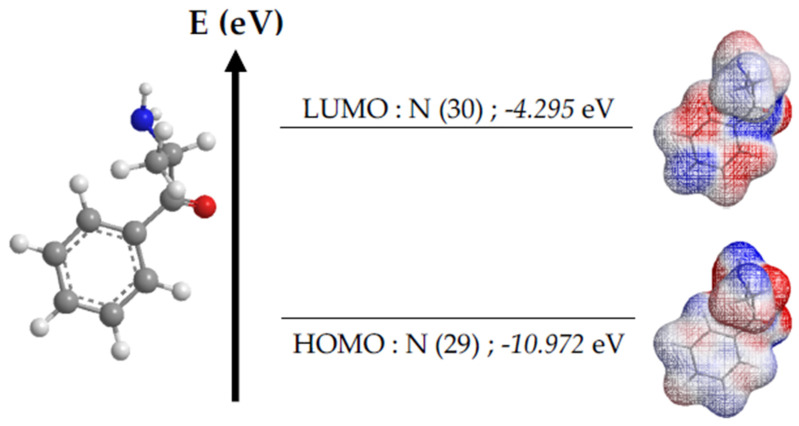
Energy level diagram of cathinone molecular orbitals.

The bioinformatics study relies on two distinct computational approaches. The first focuses on the energetic analysis of the cathinone molecule, while the second explores the molecular docking of cathinone with three proteins present in gingival tissues, playing an important role in the defense against infections. The results of the first approach are shown in Figure 6 and Table 4. Figure 6 shows the energy level diagram of the molecular orbitals of cathinone, optimized by the MMFF94 method, while Table 4 shows other calculations and its relevant energy sources. These data reveal high electronic stability, characterized by an energy gap of 6677 eV and a chemical hardness of 3338 eV, indicating increased resistance to reactivity. An electronegativity of 7634 eV suggests a strong attraction of electrons, contributing to molecular stability. The electrophilicity index of 8729 eV highlights the moderate capacity of the molecule to accept electrons during chemical reactions. Finally, the molecularity of 0.149 eV indicates moderate reactivity. In synthesis, the molecule exhibits high electronic stability, low reactivity and a moderate ability to participate as an electrophile in chemical reactions. These results are fundamental for understanding the electronic behavior and potential reactivity of the molecule studied [54,55,56]. 

**Figure 7:**
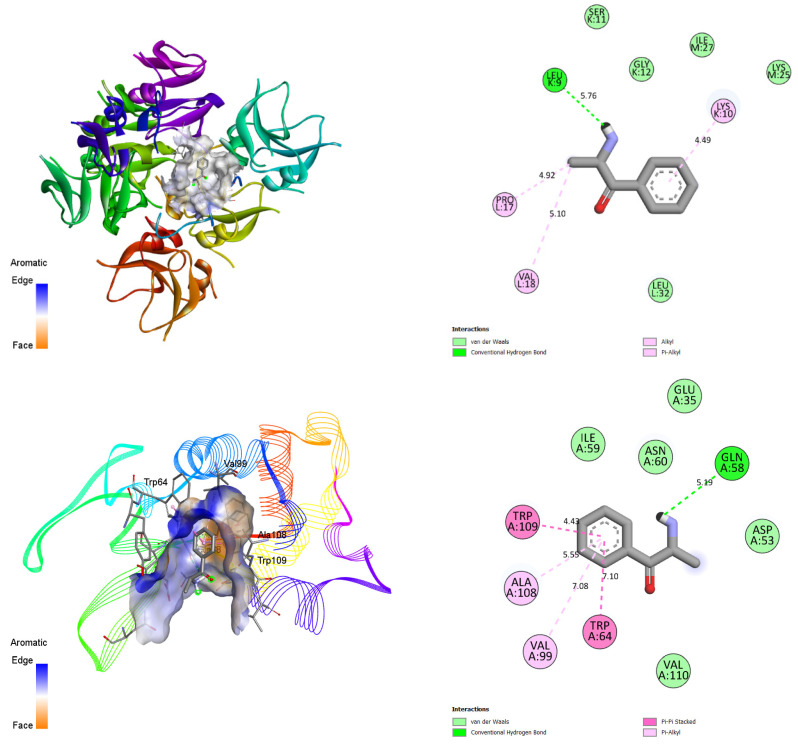

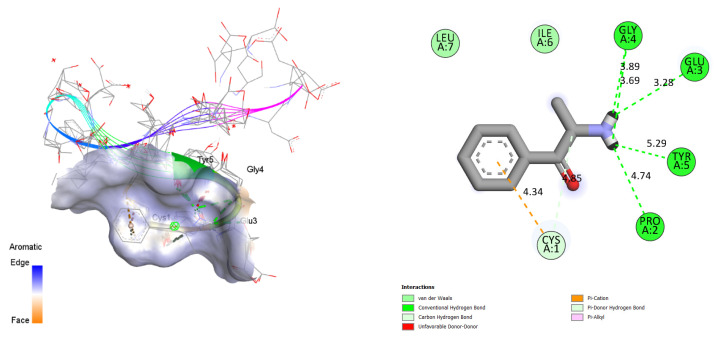
Three-dimensional and 2D docked views of cathinone with 1FD4, 1B7S and 1EGT proteins, respectively.

**Figure 8:**
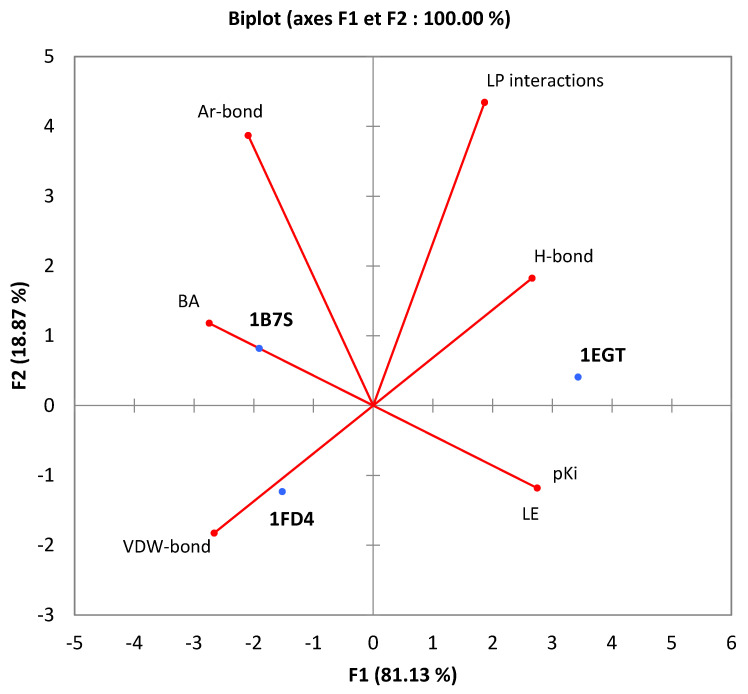
Correlation between the molecular docking parameters between cathinone and the proteins studied.

The second approach focuses on molecular docking between cathinone and three protein receptors present in gingival tissues, contributing to protection against infections. These proteins include 1FD4, an antimicrobial defensin [57], 1B7S, a lysozyme with antibacterial properties [58], and 1EGT, a thrombomodulin regulating inflammation [59]. The choice of these proteins is motivated by their enhanced role in the first line of defense against oral infections and the maintenance of a healthy oral environment. The results of this study, presented in 3D and 2D in Figure 7 and Table 5, include several quantitative and qualitative parameters such as binding affinity (BA), pKi, ligand efficiency (LE), interactions ligand–protein (LP), the number of conventional hydrogen bonds (H-Bond), the number of aromatic bonds (Ar-Bond), and the number of Van Der Waals bonds (VDW-bond). A principal component analysis (PCA), shown in Figure 8, simplifies the visualization of the information and reveals significant correlations between these parameters. Notably, 1FD4 and 1B7S proteins show a correlation with the number of aromatic bonds and the number of Van Der Waals bonds, suggesting their importance in the formation of the ligand–receptor complex [60,61]. In contrast, the 1EGT protein correlates with the number of conventional hydrogen bonds, highlighting that the attractive forces between cathinone and this receptor rely primarily on these interactions, including dispersion forces and dipole–dipole interactions [62,63,64].

## 4. Conclusions

This present work was devoted to a cross-sectional study of oral pathologies under the influence of khat chewing, where the research was explored from three major perspectives in order to offer a complete vision of periodontal problems. The first section focused on physicochemical analyses of the teeth of khat consumers, revealing the significant erosion of dental enamel due to chewing. This alteration is supported by the deterioration of the crystal structure of the main dental compounds, accompanied by a notable increase in organic matter, creating an environment conducive to the formation of pathogenic bacterial films. Furthermore, the second part explored the effects of khat on gingival plasma cell infections, highlighting alterations in the ultrastructure of enamel and suggesting negative impacts on dental health. The third part adopted a bioinformatics approach targeting cathinone, the predominant molecule of khat, and studied its interactions with crucial proteins in gingival tissues. These results enriched the understanding of the consequences of khat chewing on oral health, highlighting the importance of specific interventions to address the challenges associated with this cultural practice, thus prompting further research in this area.

The emerging perspectives of this research theme open horizons for future developments and initiatives focused on an in-depth understanding of the impacts of khat chewing on oral health. These perspectives suggest the potential for targeted pharmacological interventions, the establishment of awareness and prevention initiatives within communities where the practice of khat is widespread, as well as the promotion of multidisciplinary approaches involving collaborations between researchers, such as odontologists, biochemists and bioinformaticians, and designing holistic solutions to the specific challenges posed by khat chewing on oral health.

## Figures and Tables

**Table 1:** Equations to calculate the numerical energetic descriptors.

Descriptors	Symbol	Equation
Energy gap	*E_GAP_*	$E_{LUMO}\;-\;E_{HOMO}$
Chemical hardness	*η*	$\frac{E_{LUMO}\;-\;E_{HOMO}}{2}$
Electronegativity	*χ*	$-\left(\frac{E_{LUMO}\;+\;E_{HOMO}}{2}\right)$
Electrophilicity index	*ω*	$\frac{\chi^{2}}{2\eta }$
Molecular flexibility	*S*	$\frac{1}{2\eta }$

**Table 2:** The active site coordinates of studied proteins (1FD4, 1B7S, and 1EGT).

Protein	1FD4	1B7S	1EGT
Size (Å)	x = 40; y = 40; z = 40	x = 40; y = 40; z = 40	x = 40; y = 40; z = 40
Center (Å)	x = 7.675	x = 13.157	x = 0.199
	y = 21.015	y = 14.900	y = 0.148
	z = 18.444	z = 28.924	z = −0.114

**Table 3:** Comparison of calcium, phosphate, calcium/phosphorus ratio and microhardness of tooth surfaces of samples DK and DNK. (DK: teeth of Khat consumers, DNK: teeth of non-consumers of Khat).

Teeth	Calcium (%)	Phosphorus (%)	Ca/P Ratio	Microhardness (HV)
DK	17.45 ± 2.54	6.32 ± 1.01	2.76 ± 0.11	52.16 ± 5.64
DNK	32.87 ± 1.74	15.09 ± 1.36	2.17 ± 0.16	244.68 ± 5.79

**Table 4:** Calculated energy values of cathinone.

E_GAP_	η	χ	ω	S
6.677	3.338	7.634	8.729	0.149

**Table 5:** Energetic and qualitative characteristics of molecular docking of cathinone with the proteins studied.

Proteins	Binding Affinity (kcal.mol^−1^)	p*Ki*	Ligand Efficiency (kcal.mol^−1^)	Ligand–Protein Interactions	Number of Conventional Hydrogen Bonds	Number of Aromatic Bonds	Number of Van Der Waals Bonds
1FD4	−5.7	4.18	0.5182	4 (Leu9—Pro17—Val18—Lys10)	1	1	5
1B7S	−5.6	4.11	0.5091	5 (GLN58—TRP64—Val99—ALA108—TRP109)	1	4	5
1EGT	−9.7	7.11	0.8818	6 (GLY4—GLY4—GLU3—TYR5—PRO2—CYS1)	5	1	2

## Data Availability

Data is contained within the article.
